# Functional proteomic analysis of seminal plasma proteins in men with various semen parameters

**DOI:** 10.1186/1477-7827-11-38

**Published:** 2013-05-11

**Authors:** Rakesh Sharma, Ashok Agarwal, Gayatri Mohanty, Rachel Jesudasan, Banu Gopalan, Belinda Willard, Satya P Yadav, Edmund Sabanegh

**Affiliations:** 1 Center for Reproductive Medicine, Glickman Urological and Kidney Institute, Cleveland Clinic, Cleveland, OH, USA; 2 Center for Cellular and Molecular Biology, Hyderabad, Andhra Pradesh, India; 3 Bioinformatics Core Services, Lerner Research Institute, Cleveland Clinic, Cleveland, OH, USA; 4 Proteomics Core Services, Lerner Research Institute, Cleveland Clinic, Cleveland, OH, USA; 5 Molecular Biotechnology Core lab, Lerner Research Institute, Cleveland Clinic, Cleveland, OH, USA; 6 Permanent Address: Ravenshaw University, Cuttack, Odisha, India

## Abstract

**Background:**

Alterations at the molecular level in spermatozoa and seminal plasma can affect male fertility. The objective of this study was to determine if analysis of differential expression of proteins in varying semen parameters can serve as potential biomarkers for male infertility.

**Methods:**

The differential expression of proteins in the seminal plasma of men based on sperm count and morphology were examined utilizing proteomic tools. Subjects were categorized based on sperm concentration and morphology into 4 groups: 1) normal sperm count and normal morphology (NN); 2) normal sperm count and abnormal morphology (NA); 3) oligozoospermia and normal morphology (ON); and 4) oligozoospermia and abnormal morphology (OA). Proteomic analysis was performed by LC-MS/MS followed by functional bioinformatics analysis. Protein distribution in the NA, ON and OA groups was compared with that of the NN group.

**Results:**

Twenty proteins were differentially expressed among the 4 groups. Among the unique proteins identified, 3 were downregulated in the NA group, 1 in the ON group and 1 in the OA group while 2 were upregulated in the ON and OA groups. The functional analysis 1) identified biological regulation as the major processes affected and 2) determined that most of the identified proteins were of extracellular origin.

**Conclusions:**

We have identified proteins that are over-or underexpressed in the seminal plasma of men with poor sperm quality. The distinct presence of some of the proteins may serve as potential biomarkers and provide insight into the mechanistic role played by these proteins in male infertility. Further studies using Western Blot analysis are required to validate these findings.

## Background

Infertility is a major problem in 15% of couples worldwide. Male factors may play a role in half of these cases [1]. Most causes of male infertility are idiopathic. Semen analysis remains the cornerstone in the evaluation of male infertility. However, the data generated from this routine testing do not provide any insight into the underlying problems associated with developing spermatozoa. Sperm morphology plays an important role in conception, and both fertilization and pregnancy rates are affected when morphologically normal sperms are below 5%. It is also a reflection of poor testicular physiology and is an important factor in male infertility [2-4]. However, a significant overlap of semen parameters such as sperm count, motility and morphology have been documented [5]. Idiopathic and unexplained infertility cannot be diagnosed by routine sperm function tests [6]. Similarly, oligozoospermic men may have other underlying pathologies that may contribute to infertility. Evaluation solely based on semen analysis is insufficient to determine the fertility status of the male partner.

Spermatogenesis is a complex process that involves development of the undifferentiated germ cells into a highly specialized spermatozoon capable of fertilizing an oocyte [7]. Fertilization requires physical proximity of the spermatozoa and the oocytes. Seminal plasma composed of secretions from the testis, epididymis and male accessory glands [8] provides a favorable environment and serves as a vehicle for the spermatozoa as it travels to meet the oocyte.

Seminal plasma contains unique proteins necessary for sperm function and survival [9,10]. Seminal plasma proteins play a variety of roles—they help protect the sperm by binding to the sperm surface during ejaculation and play a key role in capacitation, acrosome reaction, and sperm-egg fusion [11,12]. They can also modulate immune response in male and female reproductive tracts, ensuring that the most competent spermatozoa meet the oocyte during fertilization [13]. Thus, seminal plasma proteins can serve as important biomarkers for male infertility [14].

Conventional 1-Dimensional gel electrophoresis studies have provided information in relation to sperm proteins and their function in normal and abnormal spermatozoa [15,16]. Advancements in mass- spectrometry and proteomic-based techniques have made it possible to analyze the complex protein mixtures found in tissues and body fluids. Several attempts have been made to identify these proteins using high-throughput techniques such as matrix assisted laser desorption ionization – time of flight (MALDI-TOF) mass spectrometry (MS) and liquid chromatography – tandem mass spectrometry (LC-MS/MS) and linear ion trap (LTQ-Orbitrap) mass spectrometry [17-21].

Alterations at the molecular level in spermatozoa and the seminal plasma may contribute to male infertility. However, even after accounting for all the advances in proteomics, there has been a great lack of detailed data in the area of comparative analysis of seminal plasma proteins associated with male infertility.

The objective of the present study was 1) to compare the differential expression of proteins in the seminal plasma from subjects with normal or abnormal sperm concentration and sperm morphology utilizing proteomic tools such as LC-MS/MS and 2) utilize the functional bioinformatics analysis to identify the cellular origin and the differentially affected processes and/or pathways of these proteins to gain insights into the mechanistic roles played by these proteins in effecting the observed phenotypes. These analyses could possibly identify potential biomarkers for male infertility.

## Methods

After obtaining Institutional Review Board approval, written consent was obtained from all subjects. Semen samples were obtained from 64 subjects who were healthy male volunteers of unproven fertility (n = 21) and men presenting to our infertility clinic for evaluation (n = 43). Semen samples were collected by masturbation after 2–3 days of sexual abstinence. Samples with leukocytospermia--a high concentration of white blood cells (>1 × 10^6^ WBC/mL)--were examined for the presence of granulocytes by the peroxidase or the Endtz test. The patients with a positive Endtz test were excluded from the study. Semen analysis was conducted according to WHO criteria as described below [22].

### Semen analysis

Following complete liquefaction (average time: 20 minutes and no more than 60 min.), manual semen analysis was performed using a MicroCell counting chamber (Vitrolife, San Diego, CA) to determine sperm concentration and percentage motility according to WHO guidelines [22]. Viability was determined with Eosin - Nigrosin stain. Smears of the raw semen were stained with a Diff-Quik kit (Baxter Healthcare Corporation, Inc., McGaw Park, IL) for assessment of sperm morphology according to WHO criteria [22].

After analysis of semen parameters, aliquots of the samples were frozen at −80°C for proteomic analysis.

### Preparation of samples for proteomic analysis

Samples were divided into 4 groups based only on normal sperm concentration and normal morphology parameters according to WHO criteria [22]. The groups were as follows: Group 1: normal sperm count and normal morphology (NN = 26); Group 2: normal sperm count and abnormal morphology (NA = 22); Group 3: oligozoospermia and normal morphology (ON = 6) and group 4: oligozoospermia and abnormal morphology (OA = 10).

To prepare the samples for proteomic analysis, they were thawed, and clear seminal plasma was separated from the sperm pellet by centrifugation at 3,000 g for 30 minutes to ensure complete removal of the cellular components. Seminal plasma samples were pooled into replicates (NN = 5; NA = 4; ON = 1; OA = 2). Each sample was dissolved in 98% acetonitrile containing 0.1% trifluoroacetic acid followed by lyophilization at −80°C under vacuum for 2 days. The lyophilized sample was used to estimate the protein content. The samples were first precipitated in cold acetone and centrifuged at 10,000 g for 15 minutes. The acetone was poured off, and the protein pellet was allowed to dry at room temperature. The protein pellet was solubilized in a buffer of 6 M urea, 100 mM Tris, pH 8.0. The proteins were then reduced by the addition of DTT (200 mM in 100 mM Tris) for 15 minutes at room temperature and then alkylated by the addition of 200 mM iodoacetamide (200 mM in 100 mM Tris) for 20 minutes at room temperature. The urea concentration was then reduced to approximately 1.2 M, and trypsin was added at a ratio of 1:50. Digestion was carried out overnight at room temperature. The digestion was stopped the next morning by adding acetic acid to lower the pH to <6, and the samples were centrifuged to remove insoluble material. The digests were then prepared for LC-MS/MS analysis by using PepClean C-18 spin columns to desalt the samples, which were then brought up in 50 μL of 1% acetic acid.

### Liquid chromatography – mass spectrometer analysis (LC-MS/MS)

The LC-MS system is a Finnigan LTQ linear ion trap mass spectrometer system. The high performance liquid chromatography (HPLC) column was a self-packed 9 cm × 75 μm (internal diameter) Phenomenex Jupiter C18 reversed-phase capillary chromatography column. Ten μL volumes of the extract were injected, and the peptides that were eluted from the column by an acetonitrile/0.1% formic acid gradient at a flow rate of 0.25 μL/min were introduced into the source of the mass spectrometer on-line. The microelectrospray ion source was set at 2.5 kV. The digest was analyzed using the data-dependent multitask capability of the instrument acquiring full scan mass spectra to determine peptide molecular weights and product ion spectra to determine the amino acid sequence in successive instrument scans [23]. This mode of analysis produces approximately 2500 collision-induced dissociation (CID) spectra of ions ranging in abundance over several orders of magnitude. The spectral count (SC) for each protein was determined. Normalized spectral count (NSC) was obtained by dividing the spectral count for each protein and the total number of spectral counts identified in the sample. The spectral counts were quantitated by taking the normalized spectral count ratio for two sets of samples. A protein was considered to be differentially expressed if there was at least a two-fold difference in the spectral count ratios between the two samples.

### Data analysis

All CID spectra collected in the experiment were used to search the National Center for Biotechnology Information (NCBI) human reference sequence database with the search engine MASCOT (Matrix Science, Boston, MA, http://www.matrixscience.com). After identification, a database consisting of all proteins identified in these searches was created and used for a second set of searches. These searches were performed with a program called SEQUEST, and the results from these SEQUEST searches were used to determine the spectral counts. Furthermore, functional bioinformatics analysis was done using publicly available software packages such as Gene Ontology annotations from GO Term Finder [24] and GO Term Mapper [25], UniProt [26], STRAP [27], and BioGPS [28]) as well as proprietary software packages (Ingenuity Pathway Analysis (IPA) from Ingenuity® Systems [29], and Metacore™ from GeneGo Inc. [30]) to identify the differentially affected processes, pathways, interactions, and cellular distribution of the proteins in the four study groups.

## Results

### Analysis of the proteins identified by LC-MS/MS

**Figure 1:**
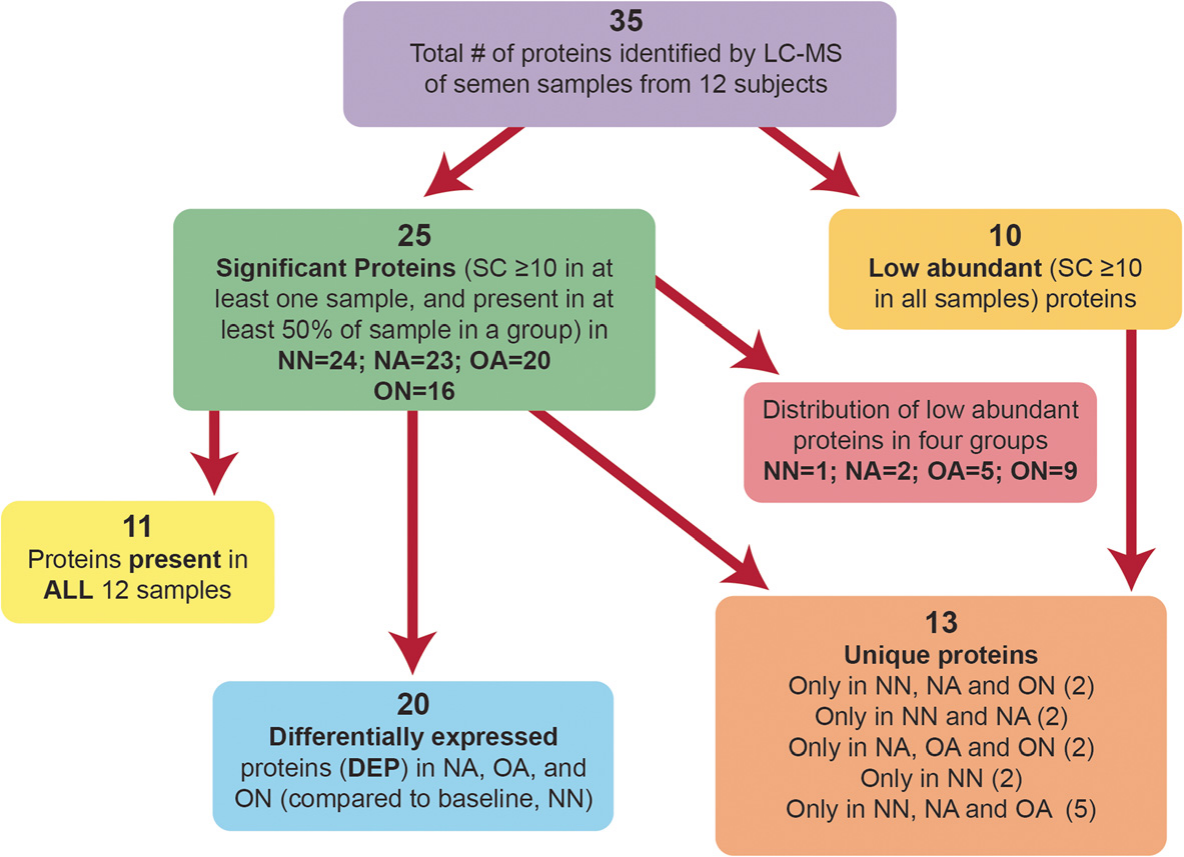
**Broad categorical analysis of proteomics data.** Differentially expressed proteins list encompasses proteins that overlap with other categories (common, unique, low abundant and significant) of proteins.

**Figure 2:**
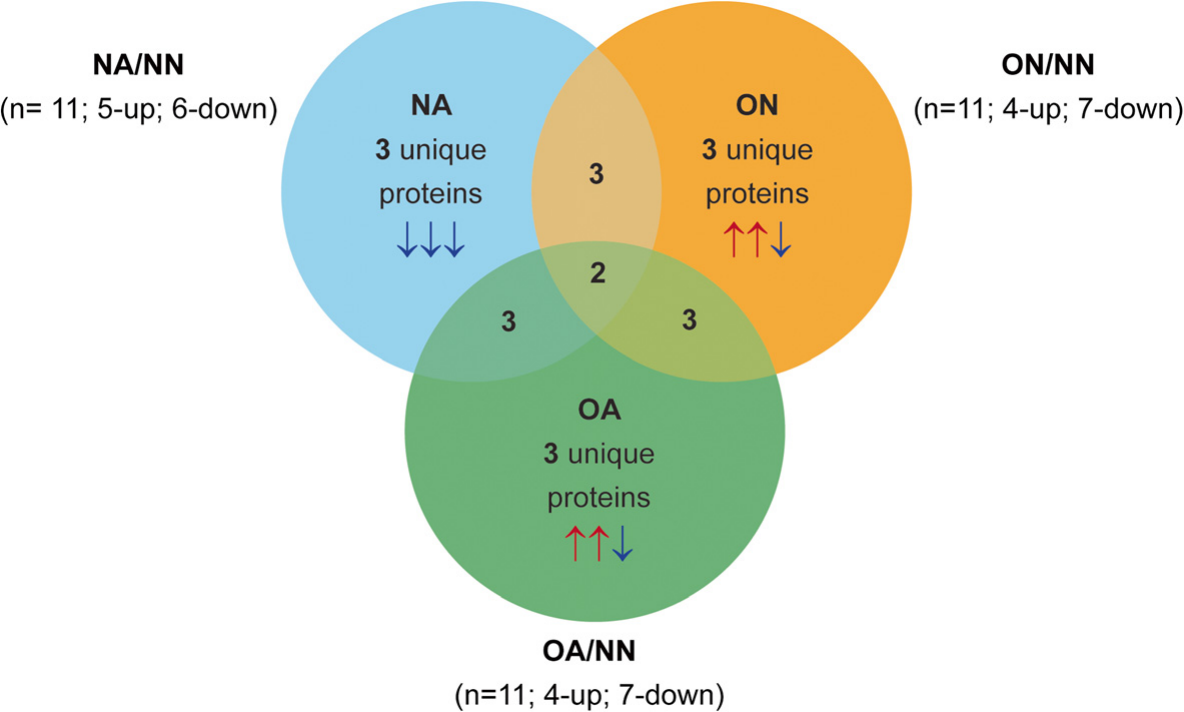
**Venn diagram showing distribution of 20 differentially expressed proteins.** This was based on the NSC ratio cut-off >2 across 3 samples, NA, OA, and ON in comparison to the baseline NN sample.

The proteins identified in the 12 replicates from NN, NA, ON and OA group showing protein name, NCBI database index, molecular weight, peptide coverage and Mascot score is shown in Table 1. A protein was considered significant if the SC cut off value was ≥ 10 in at least one sample and present in at least 50% of the samples in a group. They were considered ‘low abundant’ if the SC cut-off value was ≤10 in all the samples. The differentially expressed proteins (DEP) in the NA, OA, and ON groups were categorized based on the NSC ratio cut-off values of > 2 (for over-expressed) or < 0.5 (for under-expressed) in comparison to the NN group. We identified a total of 35 proteins; of these, 10 were classified as low abundant. Amongst the remaining 25 significantly abundant proteins (24 in NN, 23 in NA, 20 in OA, and 16 in ON), 11 were present in all the samples, and 13 proteins were identified as unique to one or two or three of the four samples. 20 proteins were identified as differentially expressed in the NA, OA, and ON groups as compared to NN group, with 2 proteins differentially expressed in all three groups (Figure 1). The remaining 18 were present in either of the groups (Figure 2). A detailed list of the proteins classified under these categories (Common, Unique, Significant, Low Abundant, and Differentially Expressed) is shown in Table 2.

**Table 1:** Identification of proteins in the 12 replicates from NN, NA, ON and OA group showing protein name, NCBI database index, molecular weight, peptide coverage and Mascot score

**Identification**				**LTQ**	
**No. Protein name**	**NCBI database index number**	**Calculated MW in kDa**	**PI**	**Peptides (%coverage)**	**Mascot score**
**Sample NN1**					
semenogelin II precursor	4506885	65	9	8(10%)	9536
prolactin induced protein	4505821	16	8.2	3(20%)	5335
albumin preproprotein	4502027	71	5.9	9(22%)	4805
epididymal secretory protein E1 precursor	5453678	16	7.5	3 (21%)	794
prosaposin isoform a preproprotein	11386147	59	5	1 (2%)	658
mucin 6, gastric	151301154	263	7.2	4 (3%)	461
prostate specific antigen isoform 4 preproprotein	71834855	24	7	3 (15%)	460
armadillo repeat protein	4502247	105	6.3	3(5%)	413
cathepsin D preproprotein	4503143	45	6.1	1 (4%)	285
zinc alpha-2-glycoprotein 1	4502337	34	5.7	2 (12%)	260
cystatin S precursor	4503109	16	4.9	2 (31%)	178
ubiquitin and ribosomal protein S27a precursor	4506713	18	9.6	1 (10%)	173
clusterin isoform 1	42716297	58	6.2	3 (10%)	142
**Sample NN2**					
prolactin-induced protein	4505821	16	8.2	10 (69%)	32886
semenogelin II precursor	4506885	65	9	17 (33%)	22468
semenogelin I isoform b preproprotein	38049014	45	9.2	13 (33%)	5388
albumin preproprotein	4502027	71	5.9	24 (54%)	15508
prostate specific antigen isoform 1 preproprotein	4502173	29	7.6	11 (61%)	5087
lactotransferrin precursor	54607120	80	8.5	22 (42%)	4281
zinc alpha-2-glycoprotein 1	4502337	34	5.7	11 (44%)	3424
cystatin S precursor	4503109	16	4.9	5 (44%)	1809
prosaposin isoform a preproprotein	11386147	59	5	9 (33%)	880
epididymal secretory protein E1 precursor	5453678	16	7.5	4 (52%)	786
serine proteinase inhibitor, clade A, member 1	50363217	46	5.3	11 (48%)	737
mucin 6, gastric	151301154	263	7.2	11 (9%)	524
extracellular matrix protein 1 isoform 1 precursor	221316614	62	6.2	7 (31%)	504
cystatin C precursor	4503107	16	9	6 (68%)	432
tissue inhibitor of metalloproteinase 1 precursor	4507509	23	8.4	4 (40%)	411
fibronectin 1 isoform 3 preproprotein	16933542	262	5.4	8(11%)	353
cathepsin D preproprotein	4503143	45	6.1	3 (11%)	266
acid phosphatase, prostate short isoform precursor	6382064	44	5.8	2 (12%)	166
carboxypeptidase E preproprotein	4503009	53	5	5 (20%)	153
clusterin isoform 1	42716297	58	6.2	2 (7%)	106
**Sample NN3**					
prolactin-induced protein	4505821	16	8.2	10 (66%)	24697
semenogelin II precursor	4506885	65	9	19 (35%)	13533
semenogelin I isoform b preproprotein	38049014	45	9.2	15 (39%)	2633
albumin preproprotein	4502027	71	5.9	30 (60%)	8842
prostate specific antigen isoform 1 preproprotein	4502173	29	7.6	16 (70%)	8634
lactotransferrin precursor	54607120	80	8.5	23 (49%)	4607
zinc alpha-2-glycoprotein 1	4502337	34	5.7	9 (42%)	3935
epididymal secretory protein E1 precursor	5453678	16	7.5	6 (52%)	1214
tissue inhibitor of metalloproteinase 1 precursor	4507509	23	8.4	3 (29%)	987
prosaposin isoform a preproprotein	11386147	59	5	5 (21%)	833
extracellular matrix protein 1 isoform 1 precursor	221316614	62	6.2	6 (22%)	766
cystatin S precursor	4503109	16	4.9	5 (49%)	621
beta 2 microglobulin precursor	4757826	13	6	2 (21%)	523
fibronectin 1 isoform 3 preproprotein	16933542	262	5.4	4 (2%)	410
orosomucoid 1 precursor	167857790	23	5	3 (16%)	397
cystatin C precursor	4503107	16	9	3 (26%)	301
mucin 6, gastric	151301154	263	7.2	7 (7%)	288
cathepsin D preproprotein	4503143	45	6.1	2 (11%)	228
carboxypeptidase E preproprotein	4503009	53	5	3 (16%)	193
acidic epididymal glycoprotein-like 1 isoform 1 precursor	25121982	29	5.5	2 (10%)	166
galectin 3 binding protein	5031863	66	5.1	4 (11%)	165
clusterin isoform 1	42716297	58	6.2	2 (7%)	158
acid phosphatase, prostate short isoform precursor	6382064	44	5.8	5 (16%)	128
prostaglandin (H2) D-isomerase −1 peptide	32171249	21	7.6	1 (8%)	115
**Sample NN4**					
prolactin-induced protein	4505821	16	8.2	12 (76%)	36052
semenogelin II precursor	4506885	65	9	16 (32%)	17746
semenogelin I isoform b preproprotein	38049014	45	9.2	14 (34%)	2723
lactotransferrin precursor	54607120	80	8.5	39 (61%)	8695
albumin preproprotein	4502027	71	5.9	23 (48%)	8361
prostate specific antigen isoform 1 preproprotein	4502173	29	7.6	15 (67%)	7417
zinc alpha-2-glycoprotein 1	4502337	34	5.7	11 (38%)	3979
mucin 6, gastric	151301154	263	7.2	14 (12%)	1685
epididymal secretory protein E1 precursor	5453678	16	7.5	7 (70%)	1428
acid phosphatase, prostate short isoform precursor	6382064	44	5.8	9 (26%)	1384
clusterin isoform 1	42716297	58	6.2	6 (16%)	1148
orosomucoid 1 precursor	167857790	23	5	4 (32%)	942
orosomucoid 2	4505529	23	5	2 (12%)	207
prosaposin isoform a preproprotein	11386147	59	5	6 (27%)	941
extracellular matrix protein 1 isoform 1 precursor	221316614	62	6.2	7 (25%)	731
tissue inhibitor of metalloproteinase 1 precursor	4507509	23	8.4	3 (29%)	635
beta 2 microglobulin precursor	4757826	13	6	2 (21%)	489
fibronectin 1 isoform 3 preproprotein	16933542	262	5.4	4 (3%)	447
cystatin C precursor	4503107	16	9	3 (26%)	391
carboxypeptidase E preproprotein	4503009	53	5	4 (14%)	358
galectin 3 binding protein	5031863	66	5.1	5 (14%)	341
transferrin	4557871	79	6.8	2 (4%)	275
acidic epididymal glycoprotein-like 1 isoform 1 precursor	25121982	29	5.5	2 (10%)	238
cystatin S precursor	4503109	16	4.9	2 (20%)	205
serine proteinase inhibitor, clade A, member 1	50363217	46	5.3	3 (12%)	187
cathepsin D preproprotein	4503143	45	6.1	2 (11%)	185
prostaglandin (H2) D-isomerase −1 peptide	32171249	21	7.6	1 (8%)	133
**Sample NN5**					
prolactin-induced protein	4505821	16	8.2	8 (44%)	15001
semenogelin II precursor	4506885	65	9	10 (14%)	7235
albumin preproprotein	4502027	71	5.9	17 (39%)	2621
epididymal secretory protein E1 precursor	5453678	16	7.5	3 (23%)	552
prostate specific antigen isoform 1 preproprotein	4502173	29	7.6	5 (22%)	539
cystatin S precursor	4503109	16	4.9	2 (23%)	332
zinc alpha-2-glycoprotein 1	4502337	34	5.7	4 (19%)	250
prosaposin isoform a preproprotein	11386147	59	5	1 (2%)	239
prostatic acid phosphatase precursor	6382064	44	5.8	2 (4%)	180
lactotransferrin	54607120	80	8.5	4 (10%)	173
clusterin isoform 1	42716297	58	6.2	2 (7%)	157
galectin 3 binding protein	5031863	66	5.1	2 (4%)	152
extracellular matrix protein 1 isoform 1 precursor	4758236	62	6.2	1 (5%)	119
**Sample NA1**					
prolactin-induced protein	4505821	16	8.2	10 (62%)	17127
albumin preproprotein	4502027	71	5.9	36 (61%)	9157
semenogelin II precursor	4506885	65	9	22 (39%)	7469
semenogelin I isoform b preproprotein	38049014	45	9.2	19 (42%)	2622
prostate specific antigen isoform 1 preproprotein	4502173	29	7.6	14 (67%)	5287
lactotransferrin	54607120	80	8.5	19 (40%)	2696
zinc alpha-2-glycoprotein 1	4502337	34	5.7	9 (38%)	1904
epididymal secretory protein E1 precursor	5453678	16	7.5	6 (68%)	1228
extracellular matrix protein 1 isoform 1 precursor	221316614	62	6.2	6 (22%)	635
serine proteinase inhibitor, clade A, member 1	50363217	46	5.3	8 (27%)	616
tissue inhibitor of metalloproteinase 1 precursor	4507509	23	8.4	2 (19%)	487
prosaposin isoform a preproprotein	11386147	59	5	4 (15%)	475
beta 2 microglobulin precursor	4757826	13	6	2 (21%)	400
fibronectin 1 isoform 3 preproprotein	16933542	262	5.4	7 (5%)	398
cystatin C precursor	4503107	16	9	3 (26%)	340
cystatin S precursor	4503109	16	4.9	2 (20%)	261
mucin 6, gastric isoform 1	89033736	185	6.3	4 (5%)	238
prostaglandin (H2) D-isomerase	32171249	21	7.6	1 (8%)	227
prostatic acid phosphatase precursor	6382064	44	5.8	4 (8%)	189
protein tyrosine phosphatase, receptor type, sigma isoform 1 precursor	104487006	218	6.1	5 (5%)	180
transferrin	4557871	79	6.8	3 (17%)	174
clusterin isoform 1	42716297	58	6.2	4 (11%)	171
cathepsin D preproprotein	4503143	45	6.1	1 (4%)	149
galectin 3 binding protein	5031863	66	5.1	5 (14%)	101
**Sample NA2**					
prolactin-induced protein	4505821	16	8.2	12 (76%)	21197
semenogelin II precursor	4506885	65	9	18 (36%)	9137
semenogelin I isoform b preproprotein	38049014	45	9.2	13 (33%)	1777
albumin preproprotein	4502027	71	5.9	26 (55%)	7695
prostate specific antigen isoform 1 preproprotein	4502173	29	7.6	16 (67%)	4963
zinc alpha-2-glycoprotein 1	4502337	34	5.7	13 (44%)	3134
lactotransferrin	54607120	80	8.5	24 (46%)	3105
epididymal secretory protein E1 precursor	5453678	16	7.5	5 (68%)	1276
prosaposin isoform a preproprotein	11386147	59	5	7 (26%)	662
tissue inhibitor of metalloproteinase 1 precursor	4507509	23	8.4	3 (29%)	660
serine proteinase inhibitor, clade A, member 1	50363217	46	5.3	5 (20%)	612
extracellular matrix protein 1 isoform 1 precursor	4758236	62	6.2	7 (22%)	577
cystatin C precursor	4503107	16	9	3 (26%)	548
beta 2 microglobulin precursor	4757826	13	6	2 (21%)	443
prostatic acid phosphatase precursor	6382064	44	5.8	8 (18%)	410
cathepsin D preproprotein	4503143	45	6.1	5 (25%)	375
clusterin isoform 1	42716297	58	6.2	2 (7%)	308
DJ-1 protein	31543380	20	6.3	2 (26%)	279
carboxypeptidase E preproprotein	4503009	53	5	4 (19%)	278
galectin 3 binding protein	5031863	66	5.1	5 (14%)	266
cystatin S precursor	4503109	16	4.9	6 (64%)	212
CD177 molecule	110735433	47	5.6	2 (9%)	199
mucin 6, gastric isoform 1	89033736	185	6.3	3 (4%)	196
fibronectin 1 isoform 3 preproprotein	16933542	262	5.4	4 (4%)	172
secretory leukocyte peptidase inhibitor precursor	4507065	15	9.1	2 (28%)	162
cathepsin B preproprotein	4503139	38	5.8	3 (12%)	127
**Sample NA3**					
prolactin-induced protein	4505821	16	8.2	5 (37%)	6642
semenogelin II precursor	4506885	65	9	6 (9%)	5126
albumin preproprotein	4502027	71	5.9	7 (17%)	2077
prosaposin isoform a preproprotein	11386147	59	5	2 (8%)	438
mucin 6, gastric isoform 1	89033736	185	6.3	3 (2%)	269
epididymal secretory protein E1 precursor	5453678	16	7.5	2 (21%)	210
zinc alpha-2-glycoprotein 1	4502337	34	5.7	3 (24%)	203
prostate specific antigen isoform 1 preproprotein	4502173	29	7.6	3 (13%)	141
clusterin isoform 1	42716297	58	6.2	3 (10%)	117
**Sample NA4**					
prolactin-induced protein	4505821	16	8.2	10(76%)	22296
semenogelin II precursor	4506885	65	9	16 (30%)	11486
semenogelin I isoform b preproprotein	38049014	45	9.2	13 (33%)	3038
prostate specific antigen isoform 1 preproprotein	4502173	29	7.6	14 (67%)	3631
zinc alpha-2-glycoprotein 1	4502337	34	5.7	11 (38%)	3260
lactotransferrin	54607120	80	8.5	16 (45%)	2926
prosaposin isoform a preproprotein	11386147	59	5	5 (20%)	773
epididymal secretory protein E1 precursor	5453678	16	7.5	3 (39%)	533
serine proteinase inhibitor, clade A, member 1	50363217	46	5.3	4 (12%)	506
extracellular matrix protein 1 isoform 1 precursor	4758236	62	6.2	4 (17%)	454
beta 2 microglobulin precursor	4757826	13	6	2 (21%)	388
tissue inhibitor of metalloproteinase 1 precursor	4507509	23	8.4	3 (29%)	351
cystatin S precursor	4503109	16	4.9	5 (48%)	321
cystatin C precursor	4503107	16	9	2 (26%)	280
cathepsin D preproprotein	4503143	45	6.1	2 (11%)	200
cathepsin B preproprotein	4503139	38	5.8	2 (10%)	186
carboxypeptidase E preproprotein	4503009	53	5	3 (11%)	162
**Sample OA1**					
prolactin-induced protein	4505821	16	8.2	12 (77%)	22670
semenogelin II precursor	4506885	65	9	19(32%)	13764
semenogelin I isoform b preproprotein	38049014	45	9.2	15 (27%)	6586
albumin preproprotein	4502027	71	5.9	26 (51%)	7715
lactotransferrin	54607120	80	8.5	28 (54%)	5063
prostate specific antigen isoform 1 preproprotein	4502173	29	7.6	11 (64%)	3309
zinc alpha-2-glycoprotein 1	4502337	34	5.7	9 (40%)	3298
ankyrin repeat domain 11	56676397	299	6.7	3 (1%)	1056
PREDICTED: mucin 6, gastric isoform 1	89033736	185	6.3	10 (14%)	808
epididymal secretory protein E1 precursor	5453678	16	7.5	7 (70%)	635
clusterin isoform 1	42716297	58	6.2	4 (13%)	512
fibronectin 1 isoform 2 preproprotein	47132551	269	5.3	10 (7%)	503
extracellular matrix protein 1 isoform 1 precursor	4758236	62	6.2	5 (28%)	420
prosaposin isoform a preproprotein	11386147	59	5	4 (18%)	407
prostatic acid phosphatase precursor	6382064	44	5.8	9 (28%)	365
tissue inhibitor of metalloproteinase 1 precursor	4507509	23	8.4	4 (35%)	337
beta 2 microglobulin precursor – 1 peptide	4757826	13	6	1 (18%)	262
serine proteinase inhibitor, clade A, member 1	50363217	46	5.3	4 (12%)	242
transferrin	4557871	79	6.8	3 (4%)	211
cathepsin D preproprotein	4503143	45	6.1	2 (11%)	206
cystatin C precursor	4503107	16	9	5 (52%)	199
cystatin S precursor	4503109	16	4.9	6 (68%)	198
secretory leukocyte peptidase inhibitor precursor −1 peptide	4507065	15	9.1	1 (18%)	175
carboxypeptidase E preproprotein	4503009	53	5	2 (8%)	123
galectin 3 binding protein	5031863	66	5.1	3 (8%)	105
cathepsin B preproprotein	4503139	38	5.8	2 (12%)	95
expressed in prostate and testis	19923082	14	8.2	1 (10%)	86
macrophage migration inhibitory factor – 1 peptide	4505185	12	7.7	1 (9%)	84
prostaglandin (H2) D-isomerase −1 peptide	32171249	21	7.6	1 (8%)	75
**Sample OA2**					
prolactin-induced protein	4505821	16	8.2	8 (51%)	13511
semenogelin II precursor	4506885	65	9	11 (18%)	6235
albumin preproprotein	4502027	71	5.9	10 (23%)	1848
cystatin S precursor	4503109	16	4.9	3 (28%)	522
prostate specific antigen isoform 4 preproprotein	71834855	24	7	4 (25%)	354
prosaposin isoform a preproprotein	11386147	59	5	1 (2%)	324
clusterin isoform 1	42716297	58	6.2	3 (10%)	308
mucin 6, gastric isoform 1	89033736	185	6.3	2 (1%)	306
zinc alpha-2-glycoprotein 1	4502337	34	5.7	4 (17%)	291
epididymal secretory protein E1 precursor	5453678	16	7.5	3 (38%)	184
ankyrin repeat domain 11	56676397	299	6.7	3 (1%)	133
carboxypeptidase E preproprotein	4503009	53	5	2 (8%)	121
lactotransferrin precursor	54607120	80	8.5	3 (9%)	115
extracellular matrix protein 1 isoform 1 precursor	4758236	62	6.2	1 (5%)	108
galectin 3 binding protein	5031863	66	5.1	1 (2%)	98
cathepsin D preproprotein	4503143	45	6.1	2 (13%)	95
lactamase, beta isoform a	26051231	61	8.7	2 (10%)	90
prostatic acid phosphatase precursor	6382064	44	5.8	1 (4%)	78
**Sample ON**					
prolactin-induced protein	4505821	16	8.2	9 (69%)	33212
semenogelin II precursor	4506885	65	9	20 (35%)	10703
semenogelin I isoform b preproprotein	38049014	45	9.2	17 ((38%)	2722
albumin preproprotein	4502027	71	5.9	25 (52%)	9519
prostate specific antigen isoform 1 preproprotein	4502173	29	7.6	14 (62%)	6487
zinc alpha-2-glycoprotein 1	4502337	34	5.7	9 (38%)	5967
lactotransferrin precursor	54607120	80	8.5	18(40%)	3770
serine proteinase inhibitor, clade A, member 1	50363217	46	5.3	4 (15%)	1138
prosaposin isoform a preproprotein	11386147	59	5	6 (26%)	1014
epididymal secretory protein E1 precursor	5453678	16	7.5	3 (39%)	937
tissue inhibitor of metalloproteinase 1 precursor	4507509	23	8.4	2 (19%)	866
extracellular matrix protein 1 isoform 1 precursor	221316614	62	6.2	3 (12%)	637
beta 2 microglobulin precursor	4757826	13	6	2 (21%)	633
mucin 6, gastric	151301154	263	7.2	5 (5%)	407
fibronectin 1 isoform 3 preproprotein	16933542	262	5.4	2 (1%)	352
cathepsin D preproprotein	4503143	45	6.1	2 (11%)	334
cystatin C precursor	4503107	16	9	3 (26%)	320
carboxypeptidase E preproprotein	4503009	53	5	3 (11%)	172
clusterin isoform 1	42716297	58	6.2	2 (7%)	147
acid phosphatase, prostate short isoform precursor	6382064	44	5.8	3 (8%)	132
secretory leukocyte peptidase inhibitor precursor	4507065	15	9.1	2 (42%)	123
protein tyrosine phosphatase, receptor type, sigma isoform 1 precursor	104487006	218	6.1	4 (3%)	112

**Table 2:** Detailed list of classification of 35 proteins based on their presence, abundance, and differential expression

**No.**	**Protein Names**	**NCBI Accession No.**	**UniProt Accession No.**	**No. of samples**	**Proteins present in groups**	**Low abundant proteins (SC ≤ 10)**	**Differentially Expressed Proteins (in NA, OA, and ON) compared to NN**	**Significant proteins in groups**
1	prolactin-induced protein	4505821	P12273	12	NN(5), NA(4), OA(2), ON(1)			NN, NA, OA, ON
2	semenogelin II precursor	4506885	Q02383	12	NN(5), NA(4), OA(2), ON(1)			NN, NA, OA, ON
3	albumin preproprotein	4502027	P02768	12	NN(5), NA(4), OA(2), ON(1)			NN, NA, OA, ON
4	lactotransferrin	54607120	P02788	12	NN(5), NA(4), OA(2), ON(1)			NN, NA, OA, ON
5	epididymal secretory protein E1 precursor	5453678	P61916	12	NN(5), NA(4), OA(2), ON(1)			NN, NA, OA, ON
6	extracellular matrix protein 1 isoform 1 precursor	221316614	Q16610	12	NN(5), NA(4), OA(2), ON(1)			NN, NA, OA, ON
7	prosaposin isoform a preproprotein	11386147	P07602	12	NN(5), NA(4), OA(2), ON(1)			NN, NA, OA, ON
8	cathepsin D preproprotein	4503143	P07339	12	NN(5), NA(4), OA(2), ON(1)			NN, NA, OA, ON
9	prostate specific antigen isoform 1 preproprotein	4502173	Q546G3	12	NN(5), NA(4), OA(2), ON(1)		↑ in OA	NN, NA, OA, ON
10	zinc alpha-2-glycoprotein 1	4502337	P25311	12	NN(5), NA(4), OA(2), ON(1)		↑ in ON	NN, NA, OA, ON
11	clusterin isoform 1	42716297	P10909	12	NN(5), NA(4), OA(2), ON(1)	Low in ON	↓ in ON	NN, NA, OA
12	mucin 6, gastric	151301154	Q6W4X9	11	NN(4), NA(4), OA(2), ON(1)		↓ in NA	NN, NA, OA, ON
13	cystatin S precursor	4503109	P01036	11	NN(4), NA(4), OA(2), ON(1)	Low in ON	↓ in NA, OA, ON	NN, NA, OA
14	galectin 3 binding protein	5031863	Q08380	11	NN(5), NA(3), OA(2), ON(1)	Low in OA and ON	↓ in OA, ON	NN, NA
15	semenogelin I isoform b preproprotein	38049014	P04279	10	NN(3), NA(4), OA(2), ON(1)		↑ in OA	NN, NA, OA, ON
16	prostatic acid phosphatase precursor	6382064	P15309	10	NN(4), NA(3), OA(2), ON(1)	Low in ON	↓in NA, ON	NN, NA, OA
17	cystatin C precursor	4503107	P01034	9	NN(4), NA(3), OA(1), ON(1)		↓in OA	NN, NA, OA, ON
18	tissue inhibitor of metalloproteinase 1 precursor	4507509	Q6FGX5	8	NN(3), NA(3), OA(1), ON(1)		↑ in ON	NN, NA, OA, ON
19	beta 2 microglobulin precursor	4757826	P61769	8	NN(3), NA(3), OA(1), ON(1)	Low in OA	↓ in OA; Up in ON	NN, NA, ON
20	DJ-1 protein	31543380	Q99497	6	NN(2), NA(3), ON(1)		↑ in NA, ON	NN, NA, ON
21	ankyrin repeat domain 11	56676397	Q6UB99	4	NA(1), OA(2), ON(1)	Low in NA and ON	↓ in OA, ON	OA
22	orosomucoid 1 precursor	167857790	P02763	2	NN(2), NA(1)	Low in NA	↓in NA	NN
23	serine proteinase inhibitor, clade A, member 1	50363217	P01009	8	NN(3), NA(3), OA(1), ON(1)		↑ in NA,ON; ↓ in OA	NA, OA, ON
24	transferrin	4557871	Q06AH7	6	NN(2), NA(3), OA(1)	Low abundant	↑ in NA, OA	NONE
25	secretory leukocyte peptidase inhibitor precursor	4507065	P03973	5	NN(1), NA(3), OA(1)	Low abundant	↑ in NA, OA	NONE
26	ubiquitin and ribosomal protein S27a precursor	4506713	P62979	4	NN(2), NA(1), OA(1)	Low abundant	↓ in NA, OA	NONE
27	protein tyrosine phosphatase, receptor type, sigma isoform 1 precursor	104487006	Q13332	4	NN(1), NA(2), ON(1)	Low abundant	↑ in NA, ON	NONE
28	acidic epididymal glycoprotein-like 1 isoform 1 precursor	25121982	P54107	3	NN(2), NA(1)	Low abundant	↓in NA	NONE
29	prostaglandin H2 D-isomerase	32171249	P41222	5	NN(2), NA(2), OA(1)	Low abundant		NONE
30	cathepsin B preproprotein	4503139	P07858	6	NN(3), NA(3)	Low abundant		NONE
31	expressed in prostate and testis	19923082	Q8WXA2	4	NN(2), NA(1), OA(1)	Low abundant		NONE
32	orosomucoid 2	4505529	P19652	1	NN(1)	Low abundant		NONE
33	CD177 molecule	110735433	Q8N6Q3	3	NA(1), OA(1), ON(1n with (1))	Low abundant		NONE
34	carboxypeptidase E preproprotein	4503009	P16870	9	NN(3), NA(3), OA(2), ON(1)	Low in OA and ON		NN, NA
35	fibronectin 1 isoform 2 preproprotein	47132551	P02751	10	NN(4), NA(4), OA(1), ON(1)	Low in ON		NN, NA, OA

NN = normal sperm count and normal morphology; NA = normal sperm count and abnormal morphology; ON = oligozoospermia and normal morphology; OA = oligozoospermia and abnormal morphology; number in the parenthesis indicates the number of samples.

### Identification of the common proteins

Our analyses revealed a set of 11 proteins that were common to all the samples in the 4 groups (Table 2). Prolactin induced protein (PIP), semenogelin II (SgII) precursor, albumin preprotein, lactotransferrin, epididymal secretory protein E1 precursor, extracellular matrix protein 1 isoform 1 precursor, prosaposin isoform A preprotein, cathepsin D preprotein, prostate specific antigen isoform 1 preprotein, zinc alpha-2 glycoprotein 1, and clusterin isoform 1 were the common proteins identified.

### Identification of differentially expressed proteins

As shown in Figure 1, the DEP list encompassed proteins that overlapped with other categories (common, unique, low abundant and significant). The common proteins that were also differentially expressed included prostate specific antigen isoform I preprotein; zinc alpha-2-glycoprotein 1 and clusterin isoform 1. Five low abundant proteins (transferrin; secretory leukocyte peptidase inhibitor precursor; ubiquitin and ribosomal protein S27a precursor; protein tyrosine phosphatase receptor type, sigma isoform 1 precursor, and acidic epididymal glycoprotein-like 1 isoform 1 precursor) were included as differentially expressed because their NSC ratio comparison met the 2-fold cutoff criteria. Of the 20 differentially expressed proteins, mucin 6, gastric; orosomucoid 1 precursor and acidic epididymal glycoprotein-like isoform 1 precursor were unique proteins that were down regulated in the NA group. The proteins identified as unique and upregulated in the OA group were: prostate specific antigen isoform 1 preprotein; semenogelin I isoform b preprotein. Cystatin C precursor was found to be downregulated in the OA group. The ON group showed an upregulation of two unique proteins (zinc alpha-2 glycoprotein 1 and tissue inhibitor of metalloproteinase 1 precursor); while clusterin 1 was downregulated in this group.

Some unique proteins were absent in some of the groups but were differentially expressed in other groups. These proteins included the DJ-1 protein, which was absent in the OA groups, whereas the ankyrin repeat domain 11 was absent in the NN group. Also included in this category was orosomucoid 1 precursor, which was found in significant abundance in the NN samples but in low abundance in NA sample.

### Identification of the low abundant proteins

Many of the identified proteins were low abundant proteins (SC ≤10) (Table 2). Some of these proteins were restricted to a particular group while they were absent in other groups. Transferrin, secretory leukocyte peptidase inhibitor precursor, ubiquitin and ribosomal protein S27 a precursor, prostaglandin H2 D isomerase were some of the proteins that were absent in ON group. The CD177 molecule was absent only in the NN group; while orosomucoid 2 was present only in the NN. Protein tyrosine phosphatase, receptor type, sigma isoform 1 precursor and acidic epididymal glycoprotein - like 1 isoform 1 precursor protein were absent in the OA group.

### Cellular distribution and significant biological processes for proteins in four groups

**Figure 3:**
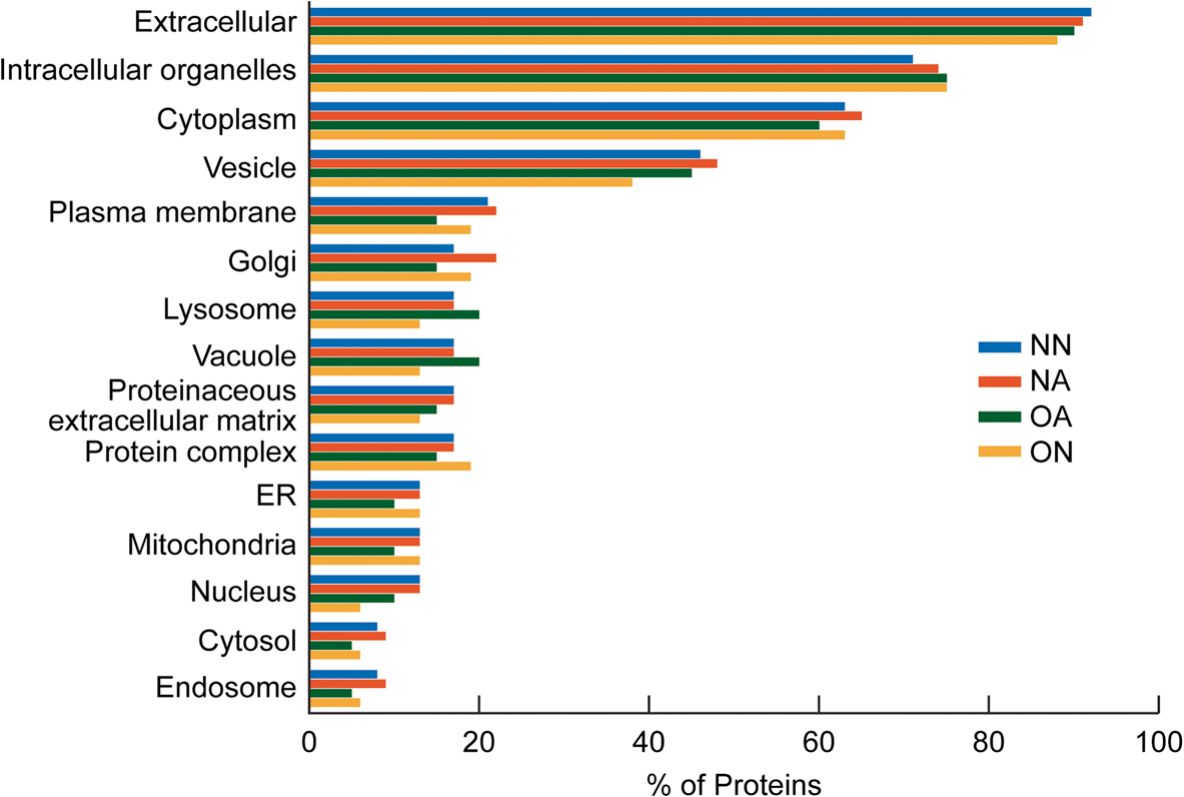
**Functional annotations from consolidated findings using publicly available software tools (GO term mapper, GO term finder, UniProtKB, STRAP, BioGPS) and proprietary pathway software packages (Ingenuity Pathway Analysis and Metacore™) showing cellular distribution of significant proteins in NN, NA, OA, and ON groups.**

The functional analysis revealed that most of the significant proteins in each of the four groups (NN, NA, OA, and ON) had a predominant cellular distribution in the extracellular region followed by their presence in the intracellular organelles (Figure 3). The distribution of proteins in the NA group was comparable in most of the cases to the NN group but was different from the ON and OA groups. The OA group showed only ~15% of the proteins localized in the plasma membrane region compared to the other groups, with the maximum number of proteins (~20%) localized in the lysosomal and vacuolar regions. The ON group showed the least distribution of proteins in the nuclear region compared to the extracellular region.

**Figure 4:**
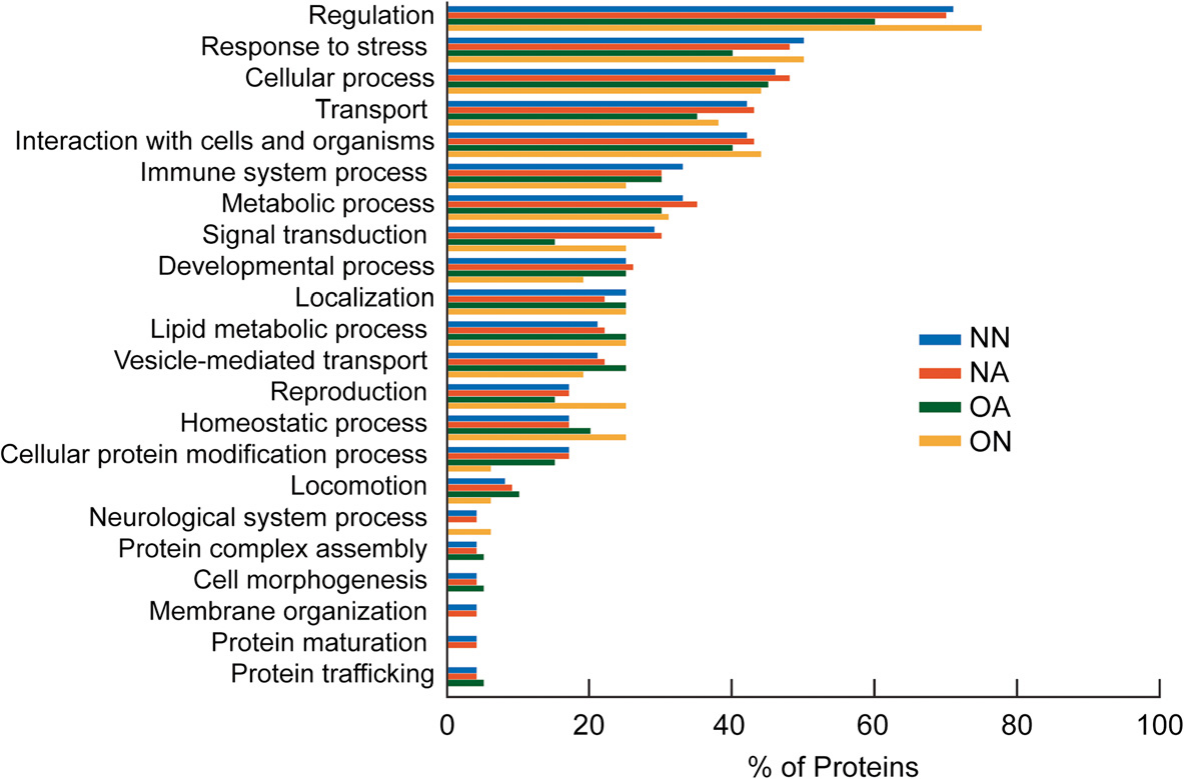
**Functional annotations from consolidated findings using publicly available software tools (GO term mapper, GO term finder, UniProtKB, STRAP, BioGPS) and proprietary pathway software packages (Ingenuity Pathway Analysis and Metacore™) showing biological processes of significant proteins in NN, NA, OA and ON group.**

The functional analysis of the significant proteins in each of the groups revealed that most of the proteins involved in the biological process were regulatory in function (Figure 4). Based on the distribution pattern of the regulatory proteins, the OA groups showed the least involvement of proteins (60%) in regulation compared with 70% - 75% seen in the NN, NA and ON groups. A smaller number of proteins were involved in other functional processes such as protein complex assembly, cell morphogenesis, membrane organization, protein maturation and trafficking in all the 4 groups. Interestingly, none of the proteins in the ON group were involved in any of these processes. Importantly, of all the major processes represented, the OA groups showed the lowest distribution of signal transduction proteins (15%) and had little or no role in neurological system processing, membrane organization and protein maturation.

### Comparison of cellular distribution and biological processes amongst the common, DEP, and low abundant proteins

**Figure 5:**
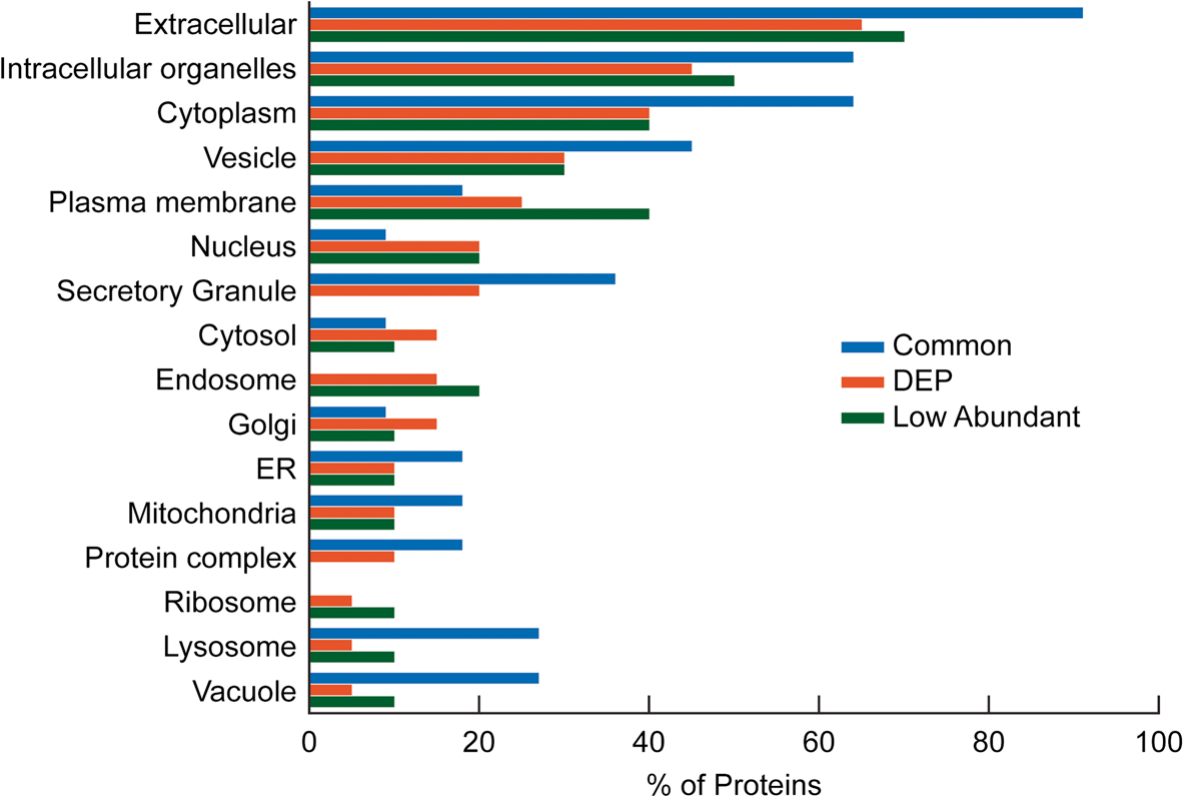
**Functional annotations from consolidated findings using publicly available software tools (GO term mapper, GO term finder, UniProtKB, STRAP, BioGPS) and proprietary pathway software packages (Ingenuity Pathway Analysis and Metacore™) showing comparison of cellular distribution amongst the proteins in Common, DEP, and Low abundant category.**

A detailed evaluation of the cellular localization of the common, DEP and low abundant proteins is shown in Figure 5. A higher distribution of the common proteins was seen in the majority of cellular compartments compared to DEP and low abundance proteins. The extracellular region showed the highest distribution (91%) of the common proteins whereas they were absent in the ribosomal and endosomal regions. Higher distribution of differentially expressed proteins was seen in the cytosolic and Golgi regions compared to the common or low abundance proteins. The low abundant proteins were absent in the protein complex and secretory granular region but their localization was found to be high in the nuclear and endosomal regions.

**Figure 6:**
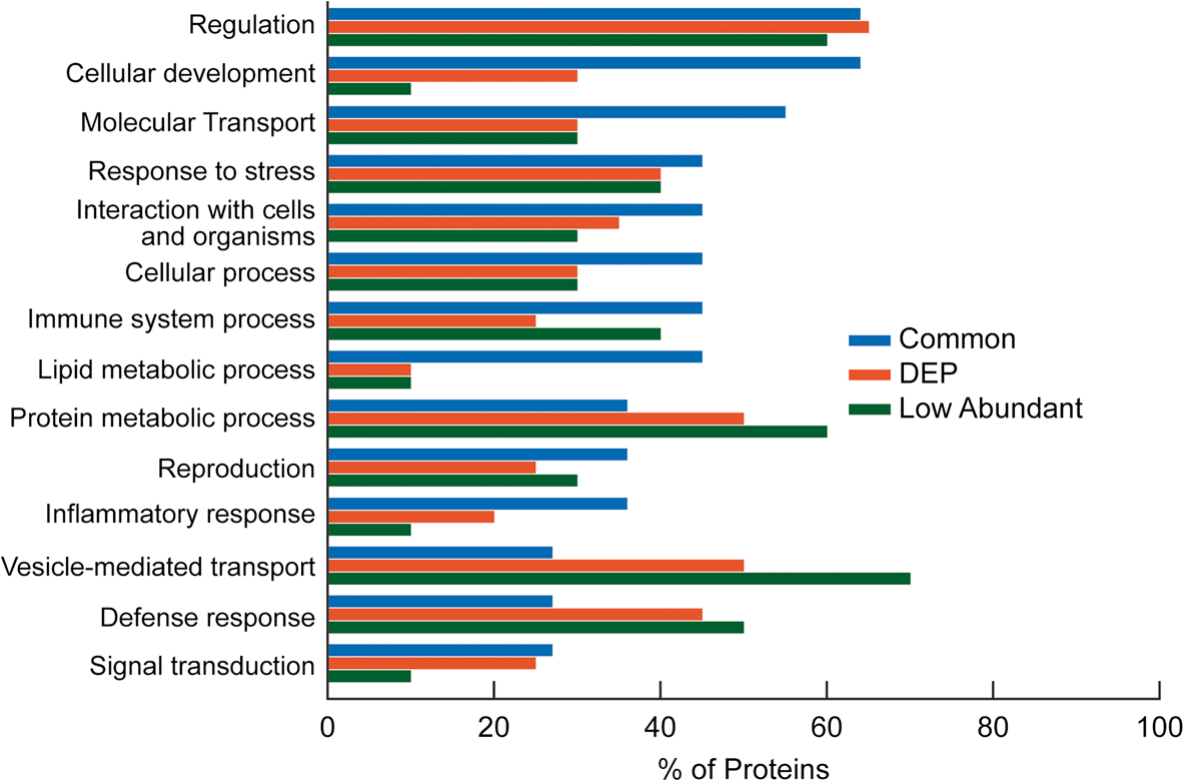
**Functional annotations from consolidated findings using publicly available software tools (GO term mapper, GO term finder, UniProtKB, STRAP, BioGPS) and proprietary pathway software packages (Ingenuity Pathway Analysis and Metacore™) showing Comparison of Biological Processes amongst the proteins in Common, DEP, and Low abundant category.**

A comparative analysis of the proteins involved in various biological processes in the three groups are shown in Figure 6. The proteins commonly expressed in all 4 groups played a significant role in many of the biological processes such as cellular development, molecular transport, and stress response, interactions with cells and organisms, cellular processes and in processes relating to the immune system. Compared with the common proteins, DEP were comparable for the regulatory processes, but were reduced in all biological processes. Higher distribution was seen in protein metabolic process, vesicle mediated transport, defense response and cellular protein modification process. The low abundant proteins were seen to be involved mainly in protein metabolism, vesicle-mediated transport and defense response.

### Pathways and network analysis using IPA and metacore™

Based on the dataset derived from common, DEP and low abundance proteins, pathways, biological functions and networks of interactions were derived utilizing the two proprietary pathway packages, IPA and Metacore™. The important processes affected by common proteins were lipid metabolism (epididymal secretory protein E1 precursor, prosaposin isoform A preprotein, clusterin isoform 1, lactotransferrin and cathepsin D preprotein), cell death and survival (cathepsin D preprotein, lactotransferrin, clusterin isoform 1, prosaposin isoform A preprotein and prostate specific antigen isoform 1 preprotein), and cellular development (clusterin isoform 1, prostate specific antigen isoform 1 preprotein, lactotransferrin, extracellular matrix protein 1 isoform 1 precursor, cathepsin D preprotein and epididymal secretory protein E1 precursor).

The important processes affected by DEP showed a higher involvement in carbohydrate metabolism and nephrosis (ankyrin repeat domain 11, beta 2 microglobulin (B2MG) precursor, clusterin isoform 1, cystatin C precursor, prostate specific antigen isoform 1 preprotein, DJ-1 protein, protein tyrosine phosphatase, receptor type, sigma isoform 1 precursor, transferrin). The major pathways involved are proteolysis (extracellular matrix; ECM), remodeling and connective tissue degradation, immune response, clathrin-mediated endocytosis signaling, lipid antigen presentation by CD1, and intrinsic prothrombin activation pathway. Similarly, in the low abundant proteins the top biological functions included the cellular development, growth proliferation, DNA replication, recombination, and repair (prostaglandin (H2) D-isomerase, protein tyrosine phosphatase, receptor type, sigma isoform 1 precursor and transferrin).

We also studied the major biological functions of the DEP in each of the 3 groups and found that free radical scavenging was the topmost function in the NA group, while cell-to-cell signaling and interaction were seen in all 3 groups. Genes that encode for 7 differentially expressed proteins (cysteine-rich secretory protein 1, clusterin, prostatic acid phosphatase (PAP), mucin 6, prostate specific antigen (PSA), zinc alpha-2-glycoprotein 1, and DJ-1) are known to be regulated by androgen receptor. The transcriptional network showed the activation of prostate induction by the androgen receptor signaling pathway. DJ-1 protein, protein tyrosine phosphatase, receptor type, sigma isoform 1 precursor and transferrin were observed to interact with other proteins in the pathway database and affect processes related to cellular function and maintenance. Similarly, in the OA group, prostate specific antigen isoform 1 preprotein and transferrin formed the topmost network, encompassing key processes such as gene expression, tissue morphology and cell cycle. The ON group showed immunological disease, antigen presentation, cell-to-cell signaling and interaction (B2MG precursor, DJ-1 protein, receptor type, sigma isoform 1 precursor) as the key processes affected in its topmost network

## Discussion

Seminal plasma is a mixture of secretions of several male accessory glands, including prostate, seminal vesicles, epididymis and Cowper’s gland. Prostate gland is a major contributor to seminal plasma. It is a very rich source of protein with concentration ranges from 35 to 55 g/l [9,10]. It provides a safe environment for spermatozoa to carry out their physiological functions. Understanding the protein profile of human seminal plasma is important because it has a profound impact on sperm physiology and thus may affect sperm functioning [31].

In this novel study, we identified 35 proteins based on SEQUEST scoring in the seminal plasma of men with varying semen parameters and categorized them into common, differentially expressed, and low abundant proteins. The large variation in the number of proteins identified by any given technique depends mainly on the sample preparation and mass spectrometry technology available [32-36]. Recently, the LTQ-Orbitrap mass spectrometer has become the cutting-edge instrument for LC/MDS/MS based approaches to characterize the seminal proteome. In our study, we used in-solution digestion of proteins with the online LC-MS system. Seminal plasma from different subsets was pooled to form 4 distinct study groups. There are numerous studies in the proteomic literature that refer to the benefits of pooling samples where it may not be feasible to analyze individual samples due to limitations of the sample or the study design [8,17,37,38].

Of the 11 proteins found in all the samples, 9 were associated with sperm function, and the common proteins comprised the majority of the secretions of the prostate gland, the seminal vesicles and epididymis. Some of the proteins or their isoforms detected in the seminal plasma were zinc alpha-2-glycoprotein 1, clusterin, lactotransferrin, prostate specific antigen. These were similar to those reported by Utleg et al. [39].

Prostate specific antigen is a serine protease that cleaves semenogelin by hydrolysis and thus liquefies the semen coagulum and facilitates sperm motility and capacitation [40,41]. Our study showed that prostate specific antigen isoform I preprotein was one of the common proteins, thus indicating its importance in all the 4 groups. Prolactin-induced protein (PIP) and Sg II are important common proteins that have a profound impact on sperm physiology. PIP has specificity to fibronectin and constitutes about 1% of seminal coagulum [42]. It may play a vital role in fibronectin breakdown during liquefaction. Viscous samples have been reported to show reduced amounts of PIP and PIP precursor, which may also be a contributory factor towards incomplete liquefaction [43]. Both Sg I and II are the major proteins of the coagulum. They represent 20-40% of the seminal plasma proteins. Studies have shown increased Sg concentrations in asthenozoospermic men [44,45]. Our study showed that both prolactin and semenogelin II were in all samples but they were not differentially expressed, indicating that men with low sperm count or abnormal morphology may not be affected by these proteins—a conclusion also made by Milardi et al. [18].

Epididymal secretory protein E I precursor, albumin preprotein, lactotransferrin, extracellular matrix protein E1 precursor, prosaposin isoform a preprotein, cathepsin D preprotein were some of the commonly expressed proteins that were not differentially expressed in any of the groups, suggesting that these proteins may not play a significant role in sperm concentration or morphology.

The highest percentage of the common proteins was seen in the extracellular region (Figure 5). Human seminal plasma proteins can bind to sperm surface proteins in the ejaculate and form a protective layer around the spermatozoon [12] as well as a sperm reservoir in the oviduct [46]. It is likely that the extracellular origin of most of the common proteins may play a key role in the binding activity of the proteins. The absence of common proteins in the ribosomal and endosomal region (Figure 5) suggests a relatively low involvement in protein metabolism, as also seen by the low distribution of common proteins in the ‘protein metabolic process’ category, (Figure 6). Zinc alpha-2 glycoprotein and clusterin play a role in signal transduction while lactotransferrin is a transport and structural protein [39]. Prostate specific antigen has been shown to have enzymatic activity. Our results show a high distribution of signal transducing protein among the common proteins, suggesting the importance of clusterin, isoform 1 and zinc alpha-2 glycoprotein 1 (Figure 6). This was also confirmed from the pathway and network analysis, which also highlighted the role these proteins play in molecular transport, cell death and survival and lipid metabolism.

A clear overlap was observed for some of the differentially expressed proteins. These included the prostate specific antigen isoform 1 preprotein, zinc alpha 2 glycoprotein 1, and clusterin isoform 1. While semenogelin II precursor was seen in common proteins, the semenogelin I isoform b preprotein was found to be upregulated in the OA group. This suggests that it may contribute to the low motility seen in this group compared to other groups.

Clusterin isoform was downregulated in the ON group. This is an interesting finding, given that clusterin isoform 1 has been shown to be downregulated in prostate cancer. The proteome of the seminal fluid is largely attributed to secretions from the prostate gland, and approximately 10% is contributed by the testis and the epididymis [8].

Prostate gland is a major contributor to seminal plasma. Furthermore, one of the proteins - prostatic acid phosphatase (PAP) was significantly increased in azoospermic men compared to oligozoospermic men and asthenozoospermic men [10]. In our study, PAP levels were down regulated in the NA and ON groups. PAP levels have also been reported to correlate with sperm concentration [47-49]. Patients with severe oligozoospermia (<1 × 10^6^) were also shown to have increased levels of seminal PAP [50]. PAP is produced in the prostate gland and is present in the seminal fluid at a concentration of 1 mg/mL. It is an important tumor marker and acts as a negative growth regulator in prostate cancer [51].

A proteomic analysis of seminal fluid, rather than blood, has been proposed as as a better starting point to identify changes that may serve as specific and sensitive markers of prostate dysfunction [17]. These authors identified more than 100 proteins such as PSA, semenogelin I and II, clusterin etc. which have been shown to affect sperm quality.

The increased expression of semenogelin I in the OA group suggests that the accessory gland secretions have a profound impact on oligozoospermic men with abnormal morphology. Wang et al. reported semenogelin I and mucin were not differentially expressed and therefore had no effect in asthenozoospermic men [21]. Our study also included the differentially expressed proteins, especially DJ-1 secreted from the testis, epididymis and prostate [39,52]. DJ-1 has a high level of expression in the testis. We found DJ-1 to be overexpressed in the NA and ON groups. We also documented that orosomucoid 1 precursor was downregulated in the NA group whereas expression of orosomucoid 2 was comparable in all groups, though it was present in low abundance. However, Wang et al. reported the overexpression of orosomucoid 1 and orosomucoid 2 in asthenozoospermic patients [21]. These proteins have also been found in high abundance in post vasectomy patients [8].

B2MG was found to be under-expressed in OA while over-expressed in the ON samples. B2MG is present in all nucleated cells. It is one of the two polypeptide chains of the major histocompatiblity complex (MHC) class I molecule. In humans B2MG is coded by the B2M gene. It is a marker of cellular immune system. It is a naturally occurring protein and can detect certain types of tumor cells in the blood and kidney, and some inflammatory and autoimmune disorders [53,54]. In our study as well as that reported by Fung et al. [17], many of the proteins in the seminal plasma were identified to be forerunners of prostate disease, suggesting a pathogenic role for B2MG [55,56]. Similarly high levels of B2MG were reported in the seminal plasma of azoospermic men compared to controls [10,57,58]. An inverse correlation was reported between B2MG levels in seminal fluid and sperm count [10].

Similary, we reported the underexpression of galectin 3 binding protein in oligozoospermic group. Galectin-3 is a carbohydrate-binding protein whose expression level has been shown to correlate with metastatic potential in a number of different types of tumors. Galectin-3 is downregulated in prostate cancer. The altered downregulation pattern of galectin-3 observed between tumor stages suggests different roles for galectin-3 in the progression of prostate cancer [59].

TIMP 1 can inhibit tumor growth, invasion and metastasis through their matrix metalloproteinases (MMP) inhibitory activity [60,61]. TIMPs play an inhibitory role, and any imbalance between the two may result in progression of the disease [62]. TIMP was found to be over-expressed in the ON group.

Amongst the proteins that were uniquely under-expressed in the OA group was ankyrin repeat domain 11 (ANKRD11). Ankyrins function as adaptor proteins [63,64] and play a vital role in membrane skeleton organization, ionic transport, maintenance of cell polarity as well as cell-cell adhesion regulation. Data suggest that ANKRD11 may act as a tumor suppressor [65]. It’s presence in the semen samples has not been well documented, but it is likely that its overexpression in oligozoospermic men may play a role in apoptosis. Clusterin isoform 1 can be both pro- and antiapoptotic depending upon the isoform expressed [66,67]. It plays a key role in signal transduction and is involved in apoptosis of spermatocytes, sperm maturation, and spermiation. Low signal transducing proteins were seen in the OA group compared with the other groups, as shown in Figure 4.

Compared with the proteins involved in stress response, as elucidated from GO annotations (Figure 4), the NA and ON groups had a higher distribution of stress proteins such as DJ-1 whereas a lower distribution of this protein was seen in OA group. DJ-1 protein is a multifunctional, highly conserved antioxidant protein and is upregulated in hyperglycemia [68,69]. It is mainly involved in the control of oxidative stress and is downregulated in the seminal plasma of asthenozoospermic men [21]. DJ-1 is activated in stress conditions, but with higher levels of stress (as seen in OA), there is depletion of antioxidant levels and low expression of DJ-1 protein. Furthermore, the major biological function that comes into focus through IPA in the NA group is free-radical scavenging activity. This further supports the fact that DJ-1 was activated in the NA group. Significant proteins were distributed in the lysosomal and vacuolar regions in higher amounts in the OA group (Figure 3), suggesting that proteins with phagocytic activity may be activated in stress conditions. Previous studies have reported reduced levels of DJ-1 in sperm in response to toxic exposure of male rats to ornidazole and epichlorhydrin [70].

Utleg et al. categorized prostatic acid phosphatase (PAP) precursor as an enzymatic protein, and the presence of this protein in our study suggests that it plays a similar role [39]. The distribution pattern of immune system response in all the groups is shown in Figure 4. It is likely that B2MG is the major protein in immune response and that low levels of this protein are seen in stress conditions. Low distribution of B2MG in DEP compared to normal and low abundant proteins further suggests that in addition to B2MG, there are other proteins that also play a key role in immune system processes. We further validated this observation from the pathway analysis, which showed that that the common protein prosaposin isoform, a preprotein, is involved in lipid antigen presentation by CD1.

We observed overlapping of differentially expressed proteins with the low abundant proteins in our study. These included transferrin, secretory leukocyte peptidase inhibitor precursor, ubiquitin and ribosomal protein S27a precursor, protein tyrosine phosphatase, receptor type, sigma isoform 1 precursor and acidic epidiymal glycoprotein- like 1 isoform 1 precursor. Of these proteins, protein tyrosine phosphatase, receptor type, sigma isoform 1 precursor, acidic epididymal glycoprotein-like isoform 1 precursor were amongst the low abundant proteins. Protein tyrosine phosphatase, receptor type, sigma isoform 1 precursor were upregulated while acidic epididymal glycoprotein-like isoform 1 precursor was down regulated in the NA group. The acidic epididymal glycoprotein like I isoform I precursor is a member of the cysteine - rich secretory protein (CRISP) family and is encoded by the gene CRISP-I. It is expressed in the epididymis and is secreted into the epididymal lumen. It binds to the post acrosomal region of the head, where it may play a role in sperm-egg fusion. The reported low abundance of acidic epididymal glycoprotein like I isoform I precursor protein is concordant with the findings of Batruch et al. who also reported its low abundance in the seminal plasma of post vasectomy patients compared to controls [8].

Transferrin is one of the serum proteins that has been characterized in the seminal plasma, but its role in male infertility is unclear [20]. Our study showed this protein was present in low levels in the NN group but was up regulated in NA and OA groups. Prostaglandin H2 D isomerase, cathepsin B preprotein, orosomucoid 2 and the CD177 molecule were the low abundant proteins that were not differentially expressed in any of the groups. In our study, CD177 was absent in the NN group but present in all the other groups although they were not differentially expressed. Our findings are consistent with previous publications that reported low concentrations of CD177 in fertile men (control samples) compared to postvasectomy men [8] but are contrary to the findings of Wang et al. who reported upregulation of CD177 in asthenozoospermic patients [21]. Cathepsin B preprotein was present in the NN and NA groups, showing its specificity to the normal sperm count while prostaglandin H2 D isomerase was absent in the ON group. Cathepsin B preprotein found in the lysosomal region is a thiol protease believed to participate in intracellular degradation. It has also been implicated in tumor invasion and metastasis. The prostaglandin (H2) D-isomerase is expressed in the testis, epididymis and prostate and is secreted into the seminal fluid. It binds small non-substrate lipophilic molecules and may act as a scavenger for harmful hydrophobic molecules. The prostaglandin (H2) D-isomerase and cathepsin B protein are potentially involved in biological processes such as vesicle-mediated transport and defense response. The low abundant proteins showed a marked distribution in the extracellular region and may play a regulatory role. The major pathways in which the low abundant proteins were involved were prostanoid biosynthesis and eicosanoid signaling and acute phase response signaling, but the distribution of signal transduction proteins was decreased in low abundant proteins.

## Conclusions

In the present study, we have identified proteins that are common, unique, and differentially expressed in 4 study groups. Twenty proteins were differentially expressed in the seminal plasma of men with poor sperm quality. We have highlighted the distribution pattern of these proteins in cell organelles and described their biological processes. We have also illustrated the high involvement of proteins in cellular development signal transduction. The overexpression or underexpression of these proteins in the 4 groups illustrates their role in male infertility. Our findings from the bioinformatic analysis shows that stress proteins such as DJ-1 are differentially regulated and expressed in the different study groups, suggesting that some of these proteins may serve as a potential biomarkers in identifying the mechanistic role in men with poor sperm quality.

## Competing interests

The authors declare that they have no competing interests.

## Authors’ contributions

RS participated in the study conception/design, review of the data and writing of the manuscript and final approval. BW, SY and BG contributed to data interpretation and participated in the paper redaction; GM participated in the review of the data and writing of the manuscript. AA contributed to the study design, and review of the data. RJ participated in organizing the subject information and review of the study findings. ES provided the subjects and participated in the study design. All authors read and approved the final manuscript.
